# Reply: Appropriate prospective trials are warranted to determine differences between exon 19 deletions and L858R EGFR mutations in non-small cell lung cancer

**DOI:** 10.1038/sj.bjc.6603565

**Published:** 2007-01-09

**Authors:** H Asahina, K Yamazaki, I Kinoshita

**Affiliations:** 1 Department of Medicine, Hokkaido University School of Medicine, North 15, West 7, Kita-ku, Sapporo 060-8638, Japan; 2 Department of Medical Oncology, Hokkaido University School of Medicine, North 15, West 7, Kita-ku, Sapporo 060-8638, Japan

**Sir**,

We would like to thank Daniel B Costa, MD for his interest in our manuscript.

In his letter, Dr Costa cited the two retrospective studies (Riely *et al*, 2006; Jackman *et al*, 2006) showing prolonged time to progression and overall survival (OS) of non-small cell lung cancer patients with exon 19 deletions when compared with L858R patients given epidermal growth factor receptor (EGFR)-tyrosine kinase inhibitors, and raised the important issue of clinical differences between these two types of mutations. As Dr Costa also mentioned, however, the two Japanese prospective trials (including our own) have shown no significant differences in response rate between these mutation types (Asahina *et al*, 2006; Inoue *et al*, 2006). In our trial, where individual data for progression-free survival (PFS) and OS were available, median PFS for exon 19 deletion was 8.3 months and two of the three patients with L858R mutation were alive and progression-free after more than 11.7 months, whereas median OS has not been reached in either group.

The lack of observed higher efficacy against exon 19 deletions compared with L858R mutations in the Japanese trials may be due to insufficient numbers of patients, as these Japanese trials were not designed to determine differences in EGFR genotypes. However, the conflicting results could also reflect ethnic differences. In addition to our tials, we calculated median PFS for each type of mutation from the retrospective data provided by Chou *et al* (2005) and Zhang *et al* (2005), as both studies were conducted in East Asian countries. Median PFS was 7.8 months in patients with exon 19 deletions and 7.6 months in patients with L858R in the former study, and 6.4 months and 10.2 months in the latter study, respectively. Although we could not calculate precise OS from those data, median PFS of patients with L858R seemed similar to or longer than that for patients with exon 19 deletions in the East Asian studies.

Conflicting results are thus apparent regarding differences in these two mutations and further data collection is required. Differences in tyrosine phosphorylation status reported from the *in vitro* study by Chen *et al* (2006) suggest the existence of clinical differences between patients with these two mutations. We hope that subsequent randomised phase III trials in different ethnic populations will answer this question.
